# Supplementary figures and images for: Cell-free chromatin particles released from dying host cells are global instigators of endotoxin sepsis in mice
Source: PLoS One. 2020 Mar 4;15(3):e0229017. doi: 10.1371/journal.pone.0229017 (PMC7055819; doi:10.1371/journal.pone.0229017)

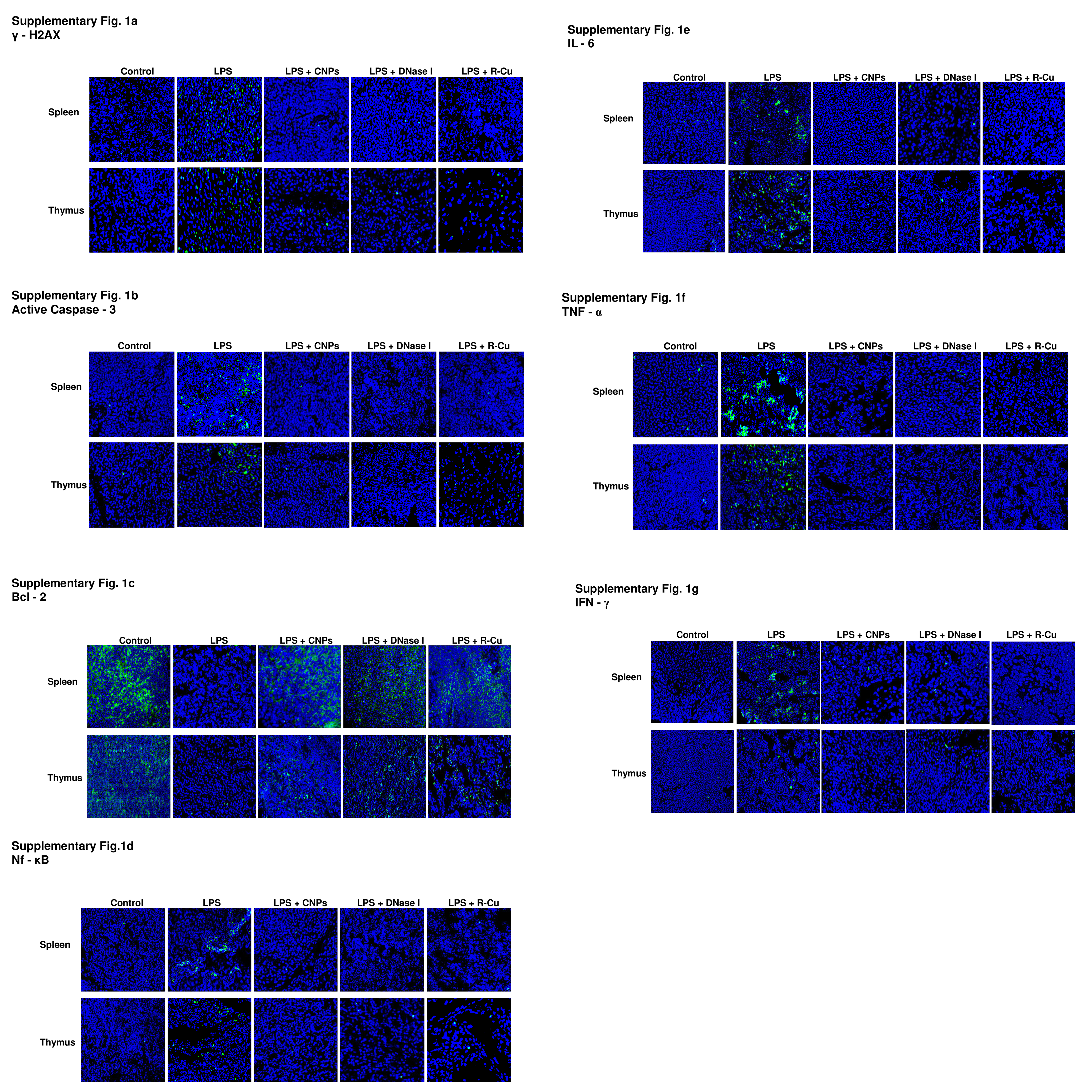

Supplement: S1 Fig — The analyses were performed at 72 h post LPS. Methodological details are given under Material and Methods section. (TIF) [file pone.0229017.s001.tif]

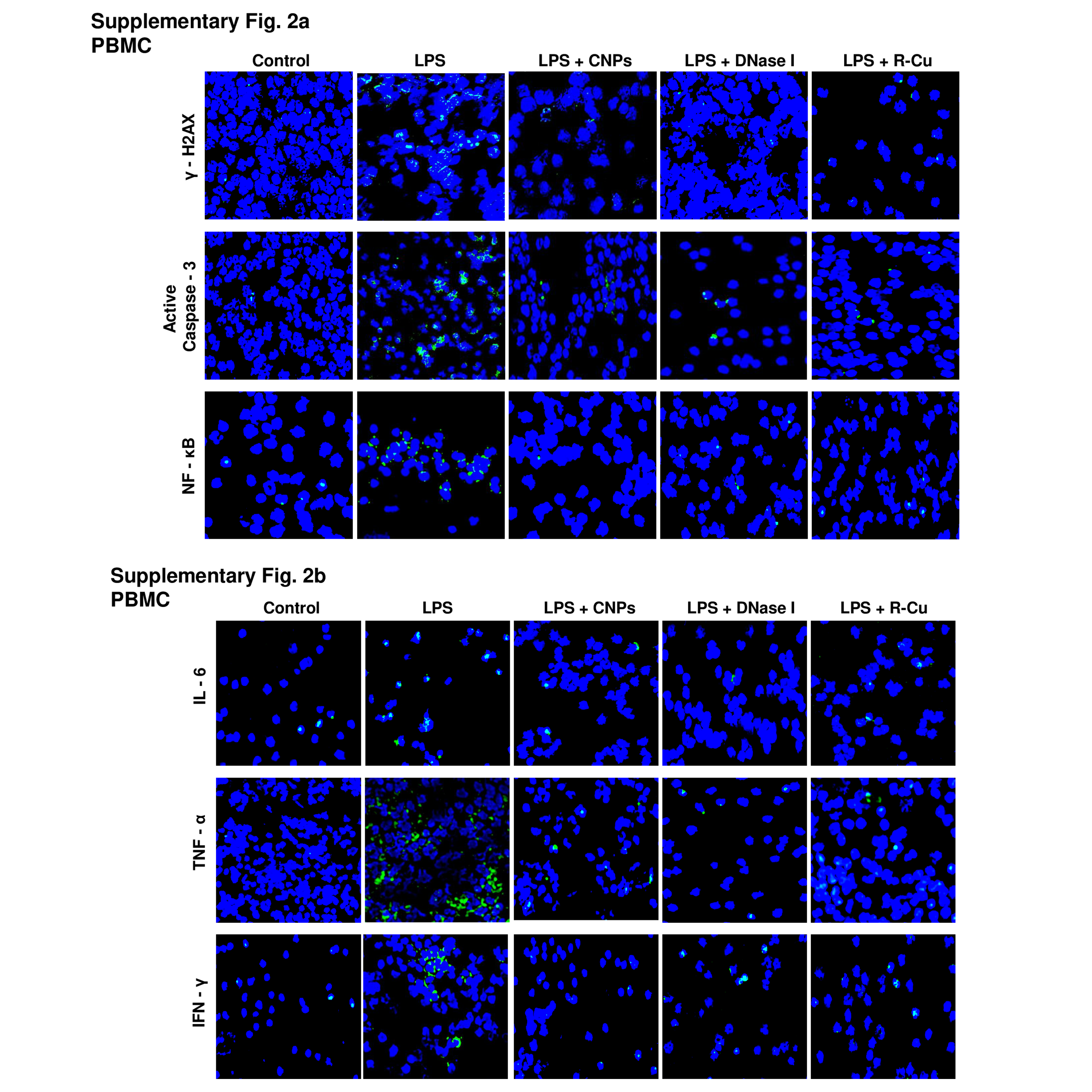

Supplement: S2 Fig — The above parameters were estimated by indirect immuno-fluorescence performed at 72 h post LPS. Methodological details are given under Material and Methods section. (TIF) [file pone.0229017.s002.tif]

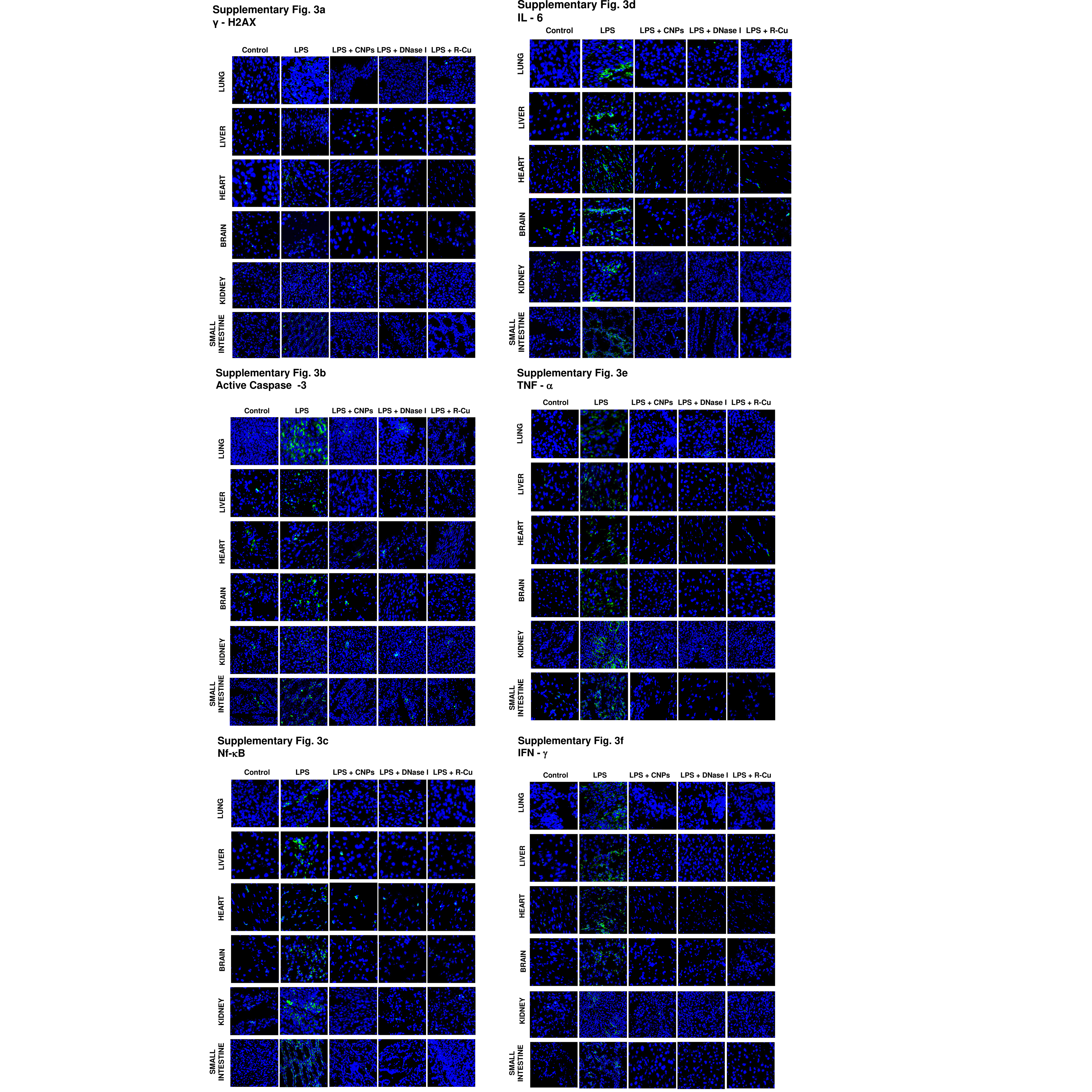

Supplement: S3 Fig — The analyses were performed at 72 h post LPS. Methodological details are given under Material and Methods section. (TIF) [file pone.0229017.s003.tif]

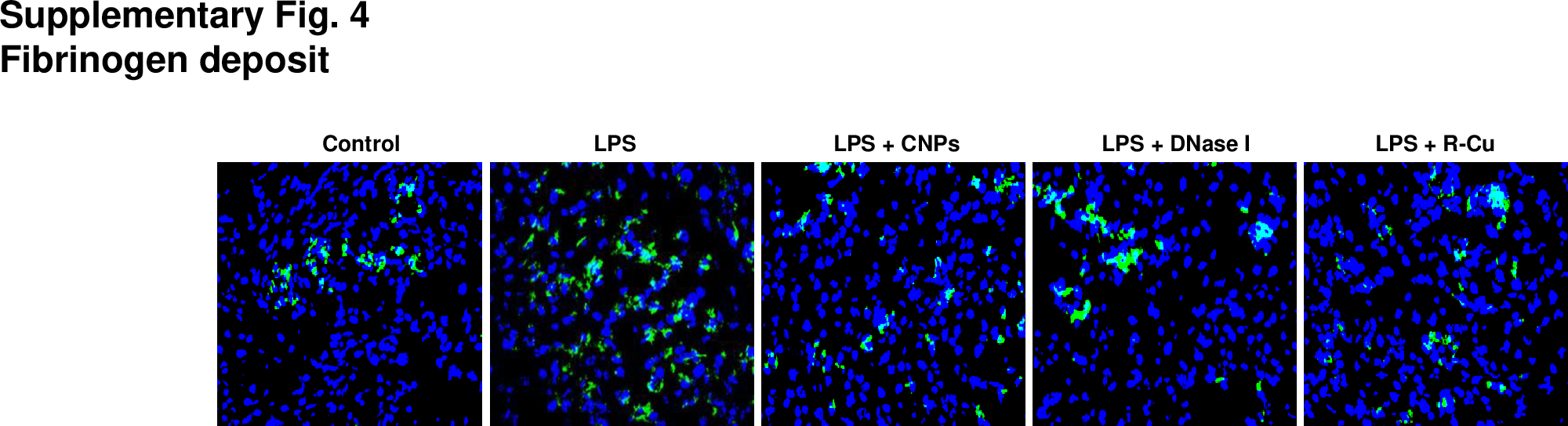

Supplement: S4 Fig — The analyses were performed at 72 h post LPS. Methodological details are given under Material and Methods section. (TIF) [file pone.0229017.s004.tif]

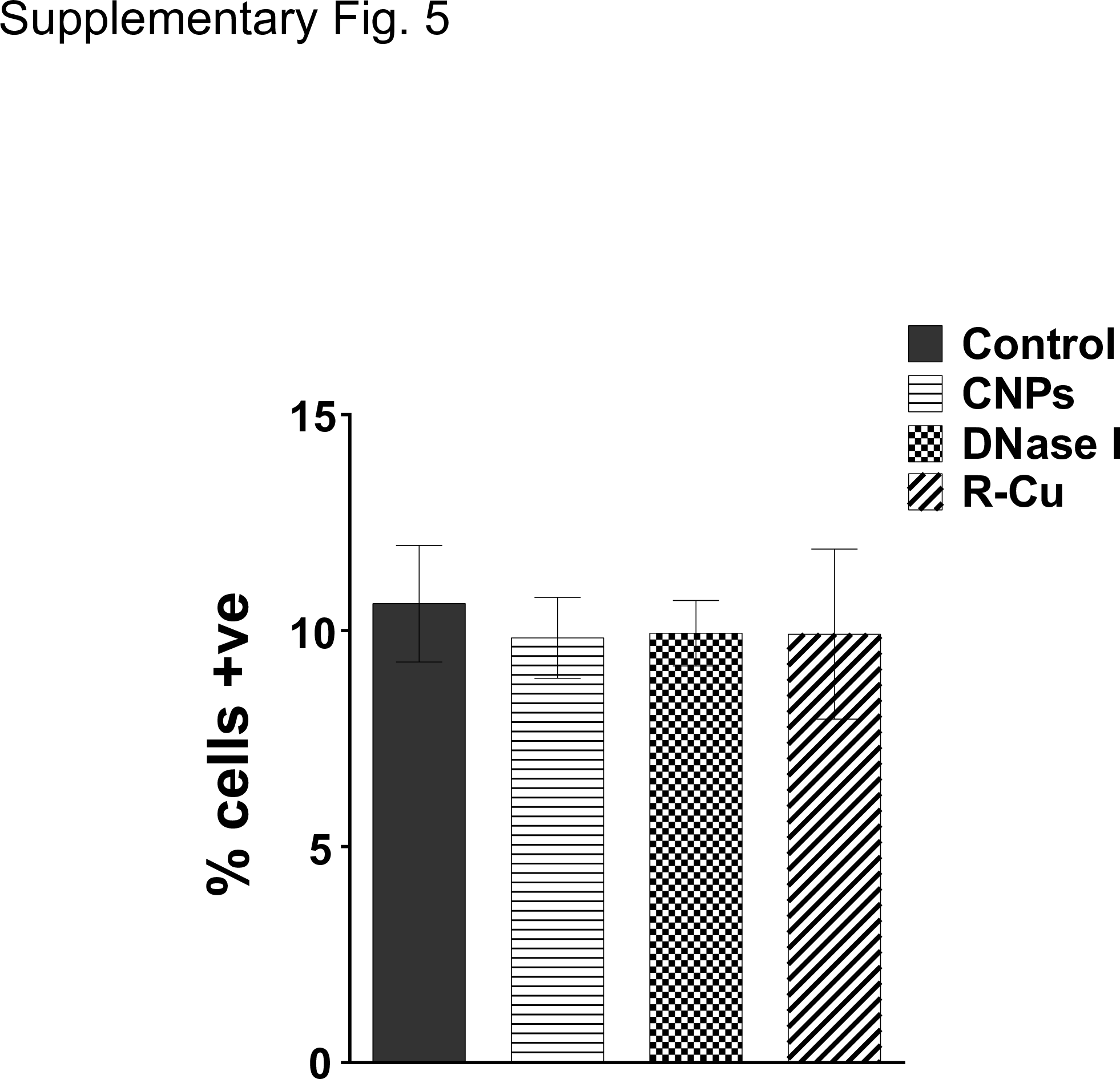

Supplement: S5 Fig — Animals were divided into four groups: 1) control (n = 10) and those receiving 2) CNPs (n = 5), 3) DNase I (n = 5) and 4) R-Cu (n = 5) in doses as described in material and methods section. Animals were sacrificed on day 7 and their brain tissues were removed and cryo-sections were prepared for estimation of γ - H2AX by immunofluorescence as described in materials and methods section. The results show that the three cfCh inactivating agents did not lead to any increase in DNA damage in terms of H2AX activation. (TIF) [file pone.0229017.s005.tif]
