# Supplementary material for: Cell-free chromatin particles released from dying host cells are global instigators of endotoxin sepsis in mice
Source: PLoS One. 2020 Mar 4;15(3):e0229017. doi: 10.1371/journal.pone.0229017 (PMC7055819; doi:10.1371/journal.pone.0229017)
Supplement: S1 Table — Results show no change in body weight during the 72h period. Changes in physical activity were not monitored in these experiments. (DOCX) [file pone.0229017.s006.docx]

**Supplementary Table 1:** Cage side parameters for assessment of body weight to evaluate side effects in experiments lasting 72 h (10 mg/kg LPS). Results show no change in body weight during the 72h period. Changes in physical activity were not monitored in these experiments.

| **Weight** | **Day 0** | **Day 1** | **Day 3** |  | **Weight** | **Day 0** | **Day 1** | **Day 3** |
| --- | --- | --- | --- | --- | --- | --- | --- | --- |
| **1^st^ Expt.**  **Control** | | | |  | **2^nd^ Expt.**  **Control** | | | |
| 1 | 20.9 | 20.8 | 20.5 |  | 1 | 21.8 | 21.8 | 21.3 |
| 2 | 22.2 | 22.2 | 21.9 |  | 2 | 21.9 | 21.9 | 21.8 |
| 3 | 21.8 | 21.8 | 21.5 |  | 3 | 22 | 22 | 21.9 |
| 4 | 22 | 22.1 | 21.9 |  | 4 | 20.9 | 20.8 | 20.6 |
| 5 | 21.9 | 21.9 | 21.6 |  | 5 | 20.9 | 20.9 | 20.9 |
| **LPS** | | | |  | **LPS** | | | |
| 1 | 20.8 | 20.8 | 20.6 |  | 1 | 20.8 | 20.6 | 20.6 |
| 2 | 21.8 | 21.8 | 21.3 |  | 2 | 20.7 | 20.7 | 20.7 |
| 3 | 22.3 | 22.2 | 21.9 |  | 3 | 20.9 | 20.9 | 20.6 |
| 4 | 22 | 22.1 | 21.9 |  | 4 | 22 | 21.9 | 21.8 |
| 5 | 21.9 | 21.9 | 21.3 |  | 5 | 21.9 | 21.9 | 21.8 |
| **LPS + CNPs** | | | |  | **LPS + CNPs** | | | |
| 1 | 21.9 | 22 | 21.9 |  | 1 | 21.9 | 21.8 | 21.7 |
| 2 | 21.3 | 21.3 | 21.4 |  | 2 | 21.8 | 21.8 | 21.6 |
| 3 | 22 | 22 | 21.9 |  | 3 | 21.4 | 21.3 | 21.3 |
| 4 | 21.9 | 21.9 | 21.8 |  | 4 | 20.9 | 20.6 | 20.4 |
| 5 | 20.8 | 20.8 | 20.6 |  | 5 | 20.8 | 20.8 | 20.7 |
| **LPS + DNase I** | | | |  | **LPS + DNase I** | | | |
| 1 | 20.7 | 20.6 | 20.4 |  | 1 | 21.9 | 21.8 | 21.4 |
| 2 | 21.8 | 21.8 | 21.6 |  | 2 | 21.8 | 21.8 | 21.7 |
| 3 | 21.8 | 21.8 | 21.4 |  | 3 | 20.6 | 20.4 | 20.3 |
| 4 | 22 | 22 | 21.8 |  | 4 | 22 | 21.9 | 21.8 |
| 5 | 21.6 | 21.6 | 21.6 |  | 5 | 20.8 | 20.7 | 20.4 |
| **LPS + R - Cu** | | | |  | **LPS + R - Cu** | | | |
| 1 | 21.6 | 21.5 | 21.4 |  | 1 | 21.8 | 21.6 | 21.4 |
| 2 | 21.8 | 21.8 | 21.6 |  | 2 | 20.9 | 20.8 | 20.6 |
| 3 | 21.9 | 21.8 | 21.4 |  | 3 | 20.9 | 20.6 | 20.3 |
| 4 | 22 | 22 | 21.8 |  | 4 | 21.3 | 21.3 | 20.9 |
| 5 | 20.7 | 20.8 | 20.6 |  | 5 | 22 | 21.9 | 21.8 |
