# Supplementary material for: Cell-free chromatin particles released from dying host cells are global instigators of endotoxin sepsis in mice
Source: PLoS One. 2020 Mar 4;15(3):e0229017. doi: 10.1371/journal.pone.0229017 (PMC7055819; doi:10.1371/journal.pone.0229017)
Supplement: S2 Table — Results show loss of body weight in LPS alone group but not in groups receiving LPS plus DNase I, R-Cu, and CNPs. B: Cage side parameters for assessment of physical activity to evaluate side effects in experiments in the lethality experiments (20 mg/kg LPS). Results show no loss of physical activity in control group. In LPs alone group loss of activity was observed. In LPS plus DNase, CNPs and R-Cu groups, a variable degree of recovery was observed. (DOCX) [file pone.0229017.s007.docx]

**Supplementary Table 2a:** Cage side parameters for assessment of body weight to evaluate side effects in the lethality experiment (20 mg/kg LPS). Results show loss of body weight in LPS alone group but not in groups receiving LPS plus DNase I, R-Cu, and CNPs.

**Supplementary Table 2b:** Cage side parameters for assessment of physical activity to evaluate side effects in experiments in the lethality experiments (20 mg/kg LPS). Results show no loss of physical activity in control group. In LPs alone group loss of activity was observed. In LPS plus DNase, CNPs and R-Cu groups, a variable degree of recovery was observed.

| **Activity assessment** | |  |  |  |
| --- | --- | --- | --- | --- |
| 0 | No activity | Not able to access food and water |  |  |
| 1 | 50 % activity | Difficulty in taking food and water |  |  |
| 2 | 100 % acitivity | Food and water easily accessible |  |  |
|  |  |  |  |  |
|  |  |  |  |  |
|  | | | | |

| **LPS** | **Day 0** | **Day 2** | **Day 4** | **Day 6** | **Day 8** | **Day 10** | **LPS + DNase I** | **Day 0** | **Day 2** | **Day 4** | **Day 6** | **Day 8** | **Day 10** |
| --- | --- | --- | --- | --- | --- | --- | --- | --- | --- | --- | --- | --- | --- |
| 1 | 2 | - | - | - | - | - | 1 | 2 | 1 | - | - | - | - |
| 2 | 2 | - | - | - | - | - | 2 | 2 | 0 | - | - | - | - |
| 3 | 2 | - | - | - | - | - | 3 | 2 | 1 | - | - | - | - |
| 4 | 2 | - | - | - | - | - | 4 | 2 | 1 | - | - | - | - |
| 5 | 2 | - | - | - | - | - | 5 | 2 | 0 | - | - | - | - |
| 6 | 2 | - | - | - | - | - | 6 | 2 | 1 | 1 | 1 | 1 | 1 |
| 7 | 2 | 0 | - | - | - | - | 7 | 2 | 1 | 1 | 1 | 1 | 1 |
| 8 | 2 | 0 | - | - | - | - | 8 | 2 | 1 | 1 | 1 | 1 | 1 |
| 9 | 2 | 0 | - | - | - | - | 9 | 2 | 1 | 1 | 2 | 2 | 2 |
| 10 | 2 | 0 | 1 | 1 | 1 | 1 | 10 | 2 | 1 | 1 | 2 | 2 | 2 |
| **LPS + CNPs** | **Day 0** | **Day 2** | **Day 4** | **Day 6** | **Day 8** | **Day 10** | **LPS + R-Cu** | **Day 0** | **Day 2** | **Day 4** | **Day 6** | **Day 8** | **Day 10** |
| 1 | 2 | - | - | - | - | - | 1 | 2 | 1 | - | - | - | - |
| 2 | 2 |  | - | - | - | - | 2 | 2 | 1 | - | - | - | - |
| 3 | 2 | 1 | - | - | - | - | 3 | 2 | 0 | - | - | - | - |
| 4 | 2 | 1 | - | - | - | - | 4 | 2 | 1 | - | - | - | - |
| 5 | 2 | 1 | - | - | - | - | 5 | 2 | 0 | - | - | - | - |
| 6 | 2 | 1 | - | - | - | - | 6 | 2 | 1 | 1 | 1 | 1 | 1 |
| 7 | 2 | 1 | 1 | - | - | - | 7 | 2 | 1 | 1 | 1 | 1 | 1 |
| 8 | 2 | 1 | 1 | 1 | 1 | 1 | 8 | 2 | 0 | 1 | 1 | 1 | 1 |
| 9 | 2 | 1 | 1 | 1 | 1 | 1 | 9 | 2 | 1 | 1 | 2 | 2 | 2 |
| 10 | 2 | 1 | 1 | 1 | 1 | 1 | 10 | 2 | 1 | 1 | 2 | 2 | 2 |
