# Supplementary material for: Cell-free chromatin particles released from dying host cells are global instigators of endotoxin sepsis in mice
Source: PLoS One. 2020 Mar 4;15(3):e0229017. doi: 10.1371/journal.pone.0229017 (PMC7055819; doi:10.1371/journal.pone.0229017)
Supplement: S3 Table — Analytical kits used and their procurement sources (Upper Table). Antibodies used and their procurement sources (Lower table). (DOCX) [file pone.0229017.s008.docx]

**Supplementary Table 3**: Analytical kits used and their procurement sources (Upper Table). Antibodies used and their procurement sources (Lower table)

| **Sr. No.** | **Kits** | **Catalogue No.** | **Source** |
| --- | --- | --- | --- |
|  | Cell Death Detection ELISA PLUS kit | REF 11920685001 | Roche Diagnostics GmbH |
|  | Mouse IL-6 ELISA kit | 550950 | BD biosciences |
|  | Mouse IL 1β ELISA kit | MLB00C | R&D Systems |
|  | Mouse IFN γ ELISA kit | 558252 | BD biosciences |
|  | Mouse TNF ELISA kit | 560478 | BD biosciences |
|  | Mouse C réactive protein ELISA kit | MCRP00 | R&D Systems |
|  | L Lactate assay | ab65331 | Abcam |
|  | Mouse Fibrinogen Simple Step ELISA Kit | ab213478 | Abcam |
|  | Mouse TAT complex ELISA Kit | ab137994 | Abcam |
|  | Antithrombin III | ab108800 | Abcam |
|  | Mouse PROC (Protein C) ELISA kit | EM1311 | Fine Test, Wuhan Fine Biotech Co. Ltd. |

| **Sr. No.** | **Antibody** | **Catalogue No.** | **Source** |
| --- | --- | --- | --- |
|  | Histone H4 IgG | Custom made | Bioklone Biotech Pvt Ltd, Chennai, India |
|  | Anti – DNA antibody | NB110-89473 | Novus Biologicals LLC, Littleton |
|  | Rhodamine labeled anti-mouse antibody | AP160R | Merck Millipore |
|  | Phospo S139 γ H2AX | ab26350 | Abcam |
|  | Anti-Caspase-3 antibody | ab2302 | Abcam |
|  | Anti-NF-kB p65 antibody | ab16502 | Abcam |
|  | Anti-IL-6 antibody | ab9324 | Abcam |
|  | IFN gamma polyclonal antobody | PA1-24782 | ThermoFisher |
|  | Mouse anti- fibrinogen γ chain Ab | Ab119948 | Abcam |
|  | FITC labeled goat anti-rabbit polyclonal antibody (active Caspase-3 secondary antibody) | AP307F | Chemicon International, Fisher Scientific |
|  | FITC labeled rabbit anti-mouse polyclonal antibody (IL-6 secondary antibody) | AP160F | Chemicon International, Fisher Scientific |
|  | FITC labeled rabbit anti-mouse polyclonal antibody (γ-H2AX secondary antibody) | AP160F | Chemicon International, Fisher Scientific |
|  | FITC Goat Anti-Rabbit (IgG) secondary antibody | ab6717 | Abcam |
|  | FITC Donkey labeled anti-goat secondary antibody | ab7121 | Abcam |
|  | FITC Goat labeled anti-mouse secondary antibody | ab6785 | Abcam |
