# Supplementary material for: Cell-free chromatin particles released from dying host cells are global instigators of endotoxin sepsis in mice
Source: PLoS One. 2020 Mar 4;15(3):e0229017. doi: 10.1371/journal.pone.0229017 (PMC7055819; doi:10.1371/journal.pone.0229017)
Supplement: Attachment — Submitted filename: Response to Reviewers 1 Jan 2020.doc [file pone.0229017.s009.doc]

**Reviewers’ Comments to the Author**

**Reviewer #1:**

**1.** First, the images cannot reflect the typical structures of brain, lung and heart tissues.

We agree. For typical structures of brain, lung and heart to be reflected we need H&E stained slides. However, in that case immunohistochemical analysis will not be possible.

Second, the DNA antibody listed by the authors is mouse IgM, but the secondary antibody is against mouse IgG. Theoretically this immunostaining cannot work.

We were aware of this. We ensured that the secondary antibody also reacted with mouse IgM. We quote from the vendor’s-Datasheet: “the secondary antibody reacts with Mouse IgG gamma chain, as well as the light chains from all Mouse immunoglobulin classes [including mouse IgM (*sic*)]” (Sigma-Aldrich, AP160R).

This has now been clarified in the text (lines 202-206, page 9).

Third, a lot of areas have positive DAPI staining but not DNA staining.

It needs to be pointed out that these are paraffin sections wherein the cut surfaces are not perfectly even. The unevenness is clearly seen in the magnified confocal images. Unevenness of paraffin sections is likely to cause the antibodies not being able to uniformly access the DNA / histone epitopes in the nuclei.

This issue has been discussed in the text (lines 307-311, page 13).

Fourth, the “positive” staining of cell free chromatin particles cannot be simply described as chromatin in the extracellular area. According to the data, they can only be described as somewhere not in the nucleus.

We agree, and we have made the necessary amendments in the text (lines 311-313 and line 316, page 13).

Fifth, the specificity of these staining, especially the possibility that they are the result of unspecific binding of antibodies to dead tissues, should be better defined.

This may be a remote possibility. We have made the necessary amendments in the text (lines 313-315, page 13).

**2**. Although the authors used three reagents to neutralize or degrade cell free chromatin, necessary controls were not used.

For neutralizing antibody, an isotype control Ig antibody should be used.

The use of an isotype control Ig antibody would have been important had we been characterizing the specificity of anti-histone antibody complexed nanoparticles (CNPs) for the first time. We are past that stage. We have already published in detail characteristics of CNPs (Ref. 19) and even obtained a US Patent (U.S. Patent No. 9,096,655, Issued: August 4, 2015). The high specificity of CNPs was fully addressed in the publication / patent application. We do not think we need to re-invent the wheel for this paper. We have also published several papers using CNPs to neutralize cfCh in mice, without Ig antibody controls, which did not evoke any queries from reviewers (Ref. 12, 13, 20, 25). We do not think it is necessary to use negative controls in a study which uses a well characterized and well established antibody complex.

For an enzyme, a deactivated enzyme should be used.

As the name suggests, DNase I degrades DNA. We do not think that it is necessary to use a deactivated enzyme as a negative control for such a specific enzyme which is universally accepted. We have also published several articles using DNase I to inactivate cfCh without any deactivated enzymes as a negative control and without any objection from reviewers (Ref. 12, 13, 20, 25).

For a compound containing heavy metal, the same organic compound with a light metal should be used.

Again, we are not addressing as to which is the best metal to use along with Resveratrol in this paper. This was done in the past when we had undertaken extensive research to come to the conclusion that Resveratrol and Copper is the best combination to degrade genomic DNA (Ref. 24), and subsequently to show that it can degrade the DNA component of cfCh to inactivate it in mice without any comments from reviewers (Ref. 12, 20, 25). During our initial extensive research we had tried out various combinations of plant poly-phenols and heavy and light metals to ultimately conclude that R-Cu was the most active combination. Light metals such as Zinc were found not to be active in combination with Resveratrol.

Nonetheless, we have now added a line to clarify that light metals are not active in combination with Resveratrol (lines 180-181, page 8).

**3**. In Figure 6, LDH is not a tissue specific parameter so the authors should not use it to define liver damage. Creatinine and BUN are kidney parameters but they are not produced by kidneys. So the authors should not put “renal” before them. Also, AST is not liver specific, so the authors should remove “liver” before AST.

We completely agree. The necessary corrections have been made in figure no. 6.

**4**. The alarmin effects of DNA, histone and other nuclear proteins have been well defined. Do the authors claim a unique function of the cell-free chromatin particles as a whole or a combined phenomenon of the already defined alarmins?

Yes, we believe that the role of cell-free chromatin (cfCh) in sepsis is unique and independent of any alarmin effects that DNA or histones might have. The sepsis inducing effects of cfCh are nullified to an equal extent by anti-histone antibody complexed nanoparticles as well as anti-DNA agents such as DNase I and R-Cu indicating that they are inactivating a common target, viz cfCh. We do not deny that free DNA and / or histones may also have independent alarmin effects; but in many of our experiments the three cfCh inactivating agents reduced the sepsis biomarkers to near control levels suggesting that cfCh is the key instigator of sepsis (Figures 3,5 and 6 for example). We have addressed this issue in the penultimate paragraph of the discussion section of the paper.

**References:** The references above pertain to those in the manuscript.

**Reviewer #2:**

**1.** Abstract (Line 29) and Introduction (Line 69): Please check sentence construction for '…those that circulated blood..'.

We thank the reviewer for pointing out these minor errors. These have now been rectified.

**2.** Introduction: It would be nice if the authors can elaborate more on the mechanisms by which cfCh particles induces DNA damage, apoptosis and activation of the inflammatory cytokine cascades. How are cfCh particles even transported from the extracellular domain into the cell? Elaborate more on findings from previous work (Refs 12-17).

Thanks are due again. This issue has now been elaborated in the introduction section (lines 73-88, Pages 3 & 4).

**3.** Materials and Methods: Animals: Please include data about any side effects experienced by the animals in the study plus their weight in the manuscript. This is important in order to rule out any harmful effects on 'bystander' healthy cells which the authors mention, but don't discuss in detail later in the discussion section.

Thanks. The issue of side effects in terms of physical activity and weight loss have now been addressed in the text (lines 131-138 page 6 and supplementary tables 1 and 2a & 2b.

With respect to harmful side effects (bystander effects), we especially undertook an experiment to assess the independent toxicity, if any, of the three cfCh inactivating agents and observed that CNPs, DNase I, and R-Cu have little toxicity of their own as assessed by activation of H2AX (DNA damage) in brain cells. The results are given in supplementary figure 5 and discussed in the text (lines 442-445, pages 18 & 19) and lines 482-483, page 20.

**4**. Materials and Methods: Detection of cellular DNA damage, apoptosis and inflammation in organs, tissues and PBMCs by IF: Please elaborate on the indirect immuno-fluorescence method used to estimate levels of gH2AX, caspase-3, Bcl-2, NF-kB, IL-6, TNFa and IFNg.

Details of indirect immuno-fluorescence method has now been given in the methods section (lines 243 – 254, pages 10 and 11).

**5.** Materials and Methods: Statistical analysis: As multiple groups are being compared simultaneously, a simple t-test won't suffice. Please use	ANOVA or a non- parametric test such as Mann Whitney.

The reviewer is absolutely right. We have reanalyzed the data using ANOVA in all our experiments. The ANOVA analytical details are given in lines 287–294, page 12.

**6**. Results: Please eliminate all figure captions. This is unnecessary, or can be used as Figure legend captions.

Figure captions have now been removed.

**7**. Results: Result titles/caption should reflect actual findings and not just what was done.
The actual findings have now been included in all figure legends.

**8**. Results: Figure 3: How were thymocytes and splenocytes isolated?

Perhaps we have created this confusion. Thymocytes and splenocytes were not isolated, rather, the various parameters were assayed on histological sections of thymus and spleen. This issue has been clarified in the text (lines 330, 335, 340 - 341, page 14).

**9.** Figures: Image quality is poor. Please upload high quality tiff images.

We have now improved the quality of the figures.

**10.** Discussion: Please discuss the potential of side effects by using all of these cfCh particle-inactivating agents on healthy cells. Elaborate on bystander effect.

In response to the reviewer’s query, we especially undertook an experiment to assess the independent toxicity, if any, of the three cfCh inactivating agents and observed that CNPs, DNase I, and R-Cu have little toxicity of their own as assessed by activation of H2AX (DNA damage) in brain cells. The results are given in supplementary figure 5 and discussed in the text (lines 442-445, pages 18 & 19) and lines 482 – 483, page 20.
