# Supplementary material for: Cell-free chromatin particles released from dying host cells are global instigators of endotoxin sepsis in mice
Source: PLoS One. 2020 Mar 4;15(3):e0229017. doi: 10.1371/journal.pone.0229017 (PMC7055819; doi:10.1371/journal.pone.0229017)
Supplement: Attachment — Submitted filename: Response to Reviewers.docx [file pone.0229017.s010.docx]

6. Review Comments to the Author

Please use the space provided to explain your answers to the questions above. You may also include additional comments for the author, including concerns about dual publication, research ethics, or publication ethics. (Please upload your review as an attachment if it exceeds 20,000 characters)

Reviewer #1: The current version of “Cell-free chromatin particles released from dying host cells are global instigators of endotoxin sepsis in mice.” made a lot of improvement compared to the last version. Here is a minor concern the authors still have to address:
The authors claimed that the effect of cell free chromatin is unique from already known alarmin effect of histone and DNA because their neutralization can bring inflammation to baseline level. However, this is not entirely true according to the result of circulating inflammatory factors (Figure 2) and sepsis survival (Figure 8). The authors need to provide a better discussion of the contribution from each inflammatory element. Also the authors need to provide some clues why tissue inflammation is resolved while circulating inflammatory factors are still high.

Our response: The reviewer’s concerns have now been addressed on page 19, lines 451-460.
